# Karyotype Patterns of *Hypsolebias antenori* (Cyprinodontiformes: Rivulidae): An Endangered Killifish of the Semiarid Region of Brazil

**DOI:** 10.1155/2014/862434

**Published:** 2014-02-11

**Authors:** Wallace Silva do Nascimento, Juliana Galvão Bezerra, Paulo Augusto Lima-Filho, Maria Emília Yamamoto, Sathyabama Chellappa, Wagner Franco Molina

**Affiliations:** ^1^Programa de Pós-Graduação em Psicobiologia, Centro de Biociências, Universidade Federal do Rio Grande do Norte, Avenida Salgado Filho 3000, Lagoa Nova, 59.072-970 Natal, RN, Brazil; ^2^Departamento de Biologia Celular e Genética, Centro de Biociências, Universidade Federal do Rio Grande do Norte, Avenida Salgado Filho 3000, Lagoa Nova, 59.072-970 Natal, RN, Brazil; ^3^Instituto Federal de Ciência e Tecnologia do Rio Grande do Norte, 59500-000 Macau, RN, Brazil

## Abstract

Annual fish which belong to the order Cyprinodontiformes constitute an excellent model for evolutionary studies. their short life cycle, distribution in ecologically dynamic environments, and low agility make them favorable for genetic analyses. The species *Hypsolebias antenori* (Rivulidae), encountered in seasonal pools located in the semiarid region of Northeastern Brazil, has been the object of surveys with a view to study its ecological and behavioral aspects. This study reports on the karyotype patterns of this species, which represents the first contribution to the cytogenetics of this genus. The karyotype of this species is composed of 2n = 48 chromosomes (6m + 4sm + 36st; NF = 96); the heterochromatic regions are located in centromeric or pericentromeric position and are more pronounced in the nucleolar organizer regions. Two sites Ag-NORs/CMA+/DAPI were identified in the short arms of pairs 2 (metacentric) and 21 (subtelocentric). Unlike the other species of this family which show an evolution modulated by events of centric fusions, *H. antenori* shows the maintenance of a basal diploid number and the large number of bibrachial elements indicates karyotypic diversification derived by pericentric inversions. Cytogenetic analyzes in this species will provide new taxonomic markers capable of being utilized in conservation issues and systematics.

## 1. Introduction

The family Rivulidae, pertaining to the order Cyprinodontiformes, is one of the largest families of freshwater fish of the Neotropical region. It is a diverse group of annual fish, most popularly known as “killifish,” which occur in seasonal freshwater pools of tropical and subtropical areas of South America. This group of fish exhibit short life cycles, thus limiting the process of sexual maturation and completion of reproductive cycle between specific seasons of a year. To survive under these extreme conditions, the fish eggs are deposited in the sediment of the pools, which go through diapause stages, during which the embryonic development becomes temporarily arrested. With the onset of the next rainy season, these eggs hatch out and a new generation is formed [1, 2]. The suborder Aplocheiloidei is represented by many species whose distribution extends over vast geographic areas covering the southeastern, central, and northeastern South America [3]. It has been suggested that, from an evolutionary point of view, that annualism in the suborder Aplocheiloidei originated and was later lost, however, it was subsequently regained [4]. Thus it demonstrates to be more plastic than it was thought previously [5].

The family Rivulidae is taxonomically composed of 30 genera and 350 valid species, with many more to be identified and described [3, 6, 7]. In the semiarid region of Brazil, they are found in the seasonal freshwater pools [2, 3]. *Hypsolebias antenori* [8] was first collected in 1945 from Ceará state in the Northeastern Brazil and was described in the genus *Simpsonichthys* [9].

A recent ecological study has included *H. antenori* in the group of endangered organisms. This species is currently under severe anthropogenic pressure, due to environmental degradation, urbanization, agricultural and ceramic production activities, and absence of conservation measures [2]. Though many species of the family Rivulidae in South America are not necessarily real evolutionary units, they demonstrate a clear pattern of morphometric and meristic differentiation [10].

Small populations are particularly subjected to constant anthropogenic changes of the environment [11]. Their biological characteristics, such as small populations, lentic habits, low vagility, and hence reduced gene flow, render them as excellent models for chromosome studies among fish.

Cytogenetic analyses in Neotropical freshwater fishes have been increasingly employed in the elucidation of taxonomic questions, phylogenetic identification of cryptic species, and delineation of populations [12, 13]. However, chromosome analysis in the Neotropical fish species of Rivulidae is scarce [14] and largely limited to species with more northerly distribution, including Colombia, Venezuela, and French Guyana [15–18], or to south of the continent covering areas of southern Brazil and Uruguay [19, 20]. Besides the deficiency of sampled species analyzed, much of the chromosome information available is mainly derived from classical cytogenetic techniques, which limit the understanding of structural aspects of the chromosomes of the species. Cytogenetic data for *H. antenori* of the semiarid regions of Northeastern Brazil are unknown.

In order to contribute to a better understanding of chromosomal evolution of Neotropical Rivulidae, this work presents cytogenetic data for the first time for a representative of the genus *Hypsolebias*. The fish species *H. antenori*, was analyzed by Giemsa staining, C-banding, silver impregnation technique of argentophilic ribosomal sites (Ag-NORs), and AT and GC base-specific fluorochromes. The results help to establish cytotaxonomic markers, which are useful as subsidies for the recognition of several evolutionary units and the biological conservation of species.

## 2. Material and Methods

**Figure 1:**
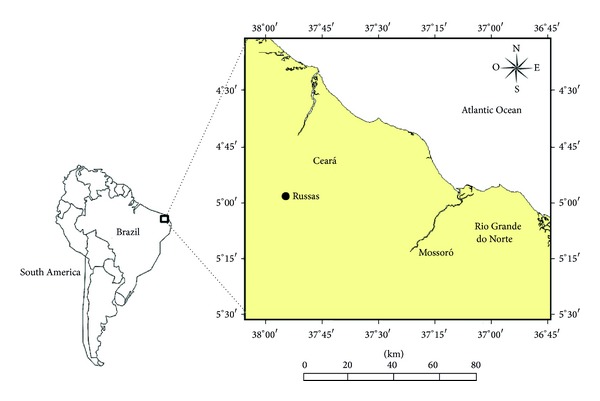
Location map showing the study area Russas in Ceará, Brazil.

Cytogenetic analyses were performed on 16 specimens of males and females of *H. antenori*, captured from temporary pools, located in the municipality of Russas, Ceará (04°57′39.8′′ S and 37°54′26.2′′ W), Northeastern Brazil (Figure 1). Fish were captured utilizing small hand trawl nets (50 × 150 cm) and sieves (60 × 60 cm) of 2 mm mesh size. The fish captured were transported to the laboratory under intense aeration. The sex was identified by macroscopic and microscopic examination of the fresh gonads. The medial portion of the gonad was placed with two drops of distilled water on a slide, covered with cover slip and lightly pressed, and immediately analyzed by an optical microscope at 200x magnification. The taxonomical identification of the fish species was verified and confirmed [8].

Adult specimens were subjected to mitotic stimulation overnight, by intraperitoneal and intramuscular injection of antigens complex, according to the methodology proposed by Molina [21] and Molina et al. [22]. After this period, the specimens were anesthetized with clove oil (1 mL/15 L water) and sacrificed for removal of the anterior kidney. Chromosome preparations were obtained by cell cycle arrest *in vitro*, according to Gold et al. [23]. The heterochromatin and nucleolar organizer regions (NORs) were identified, respectively, by using the techniques of Sumner [24] and Howell and Black [25].

In addition to the conventional staining, the chromosome preparations were also stained with chromomycin (CMA_3_) and 4′,6-diamidino-2-phenylindole (DAPI) fluorochromes [26] in order to identify regions rich in GC-or AT-, respectively. Briefly, slides were aged for three days and stained with CMA_3_ (0.1 mg/mL) for 1 h and restained with DAPI (1 *μ*g/mL), for 30 min. The slides were then mounted in glycerol : McIlvaine buffer pH 7.0 (1 : 1) and examined under epifluorescence light microscope (Olympus TM BX50), together with appropriate filters, coupled to an image capture digital system (Olympus DP73). Images of the same metaphase sequentially stained with fluorochromes CMA_3_ and DAPI were superimposed using Adobe Photoshop CS5.

The diploid number was established by analysis of thirty metaphases for each individual. The best metaphases were photographed and used in the construction of the karyotype. The chromosome morphology was determined in accordance with chromosome arms ratio [27].

## 3. Results

Males and females of *H. antenori* presented 2n = 48 chromosomes, with a karyotype composed of 6 metacentrics, 4 submetacentrics, and 36 subtelocentrics (NF = 96, number of chromosome arms). The chromosomes show little difference in size between the larger and smaller pairs and there is no evidence of structural or numerical differentiation indicative of the existence of sex chromosomes for this species.

**Figure 2:**
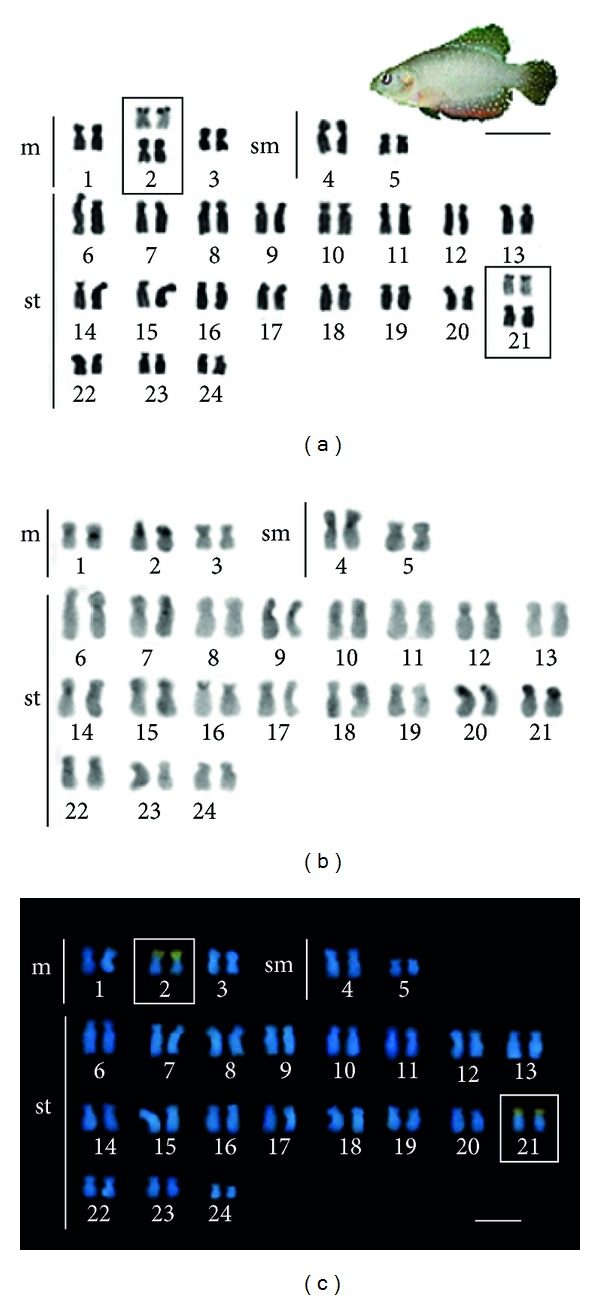
Karyotype and a male of *Hypsolebias antenori* (Bar = 1cm): (a) stained with Giemsa; (b) C-banding, and (c) sequential staining with fluorochromes CMA_3_ and DAPI. Ag-NORs sites are shown highlighted in box (bar = 5 *μ*m).

The distribution of heterochromatin in the chromosomes showed asymmetry. Thus, while few chromosome pairs (1, 2, 4, 20, 21) showed conspicuous heterochromatic blocks, in most of them, heterochromatin is reduced and is distributed preferentially in centromeric portions of chromosomes. In the pairs 2, 20, and 21 the short arms are entirely heterochromatic (Figure 2).

Analysis of double staining with CMA_3_ and DAPI fluorochromes revealed GC-rich regions, coincident with Ag-NORs sites, located on the short arms of pairs 2, metacentric, and 21, subtelocentric (Figure 2, boxes).

## 4. Discussion

Morphological features based on analyses of osteological characters, scales, color patterns, behavior, ecology, and the surface characteristics of the chorion have been used as characters useful in the diagnosis of clades in Rivulidae [28–34]. Phylogenetic analyses of South American Rivulidae species, based on molecular data, have produced inconsistent hypotheses in many cases with those based on morphological aspects [9]. Sources of additional characters may provide important insights to elucidate evolutionary aspects and kinship relations in this family.

Cytogenetic data has been widely used as a tool for understanding relationships of differentiation in groups of Neotropical fishes [12, 13, 35, 36]. In this aspect, the annual fish *H. antenori* presents a karyotype with 2n conserved, in relation to considered basal karyotype for Cyprinodontiformes (2n = 48, NF = 48) [37]. However, the high number of chromosome arms (NF = 96), involving all chromosomes of the karyotype, suggests the occurrence of evolutionary genomic reorganization for this species.

In some genera of South American Cyprinodontiformes, like *Austrolebias*, the species may present from low karyotypic diversification, as suggested by the occurrence of karyotypes with 2n = 48 and large numbers of acrocentric elements, up to more diversified, with low chromosome numbers over that considered as basic to the order, or exhibiting large structural changes evidenced by a large variation in the number of chromosome arms between species [19, 38]. Unlike other Cyprinodontiformes, *H. antenori* showed no evidence of AT-rich regions in the karyotype, as hypothesized due to intense structural rearrangements occurring in chromosomes of some species [39]. This condition, associated with the occurrence of two GC-rich regions, coincident with nucleolar organizer regions, suggests lower participation of Robertsonian translocation events, frequent in several species [15, 19, 38–40]. The chromosomal variability in annual fish corresponds to the most extreme conditions observed among the various groups of fish. The killifishes of the genus *Aphyosemion*, appear to be one of the most remarkable examples known of variability and inter-and intraspecific chromosome diversification. In this genus, reduction in the chromosome number is not entirely restricted phylogenetically [41]. This condition supports the idea suggested for other groups of fish [42], the occurrence of orthoselection karyotype process [43], where the propensity to karyotypes with Robertsonian translocations has favored its appearance multiple times through independent events. On the other hand, superposition of the cytogenetic data with molecular phylogeny available for some species of *Aphyosemion* demonstrated support to the hypothesis of Scheel [41]. In fact, as pointed out by the hypothesis, basal karyotypes, in general, have a greater number of chromosomes and high number of acrocentric chromosomes, whereas more derived species show a reduction in the number of chromosomes resulting from Robertsonian translocation events [5, 44].

More recently, extreme evolutionary dynamics involving chromosomal rearrangements has also been identified in annual African fish species of the genus *Chromaphyosemion*, where substantial cytogenetic changes related to chromosome morphology, besides banding patterns and/or diploid number between analyzed populations, were observed [39]. The karyotypic diversification among populations and species of this genus is strongly modeled by mechanisms of Robertsonian translocation, as well as other complementary processes including heterochromatinization [40]. Völker et al. [39] suggest that fixation of high cytogenetics variability in species of *Chromaphyosemion* could be due to high rate of chromosomal mutations, as well as ecological characteristics and the reproductive system of this group. In *H. antenori* pericentric inversions modulate primarily the karyotypic diversification. In this family, the pericentric inversions, along with Robertsonian translocations, represent the main mechanisms that have been operating in the karyotype evolution [15, 19].

Despite the evolutionary dynamics that exist among the killifishes, *H. antenori* appears to have a karyotype with predominantly basal traits, highlighted by the diploid number and frequency of major ribosomal genes. Yet for all, the karyotype composed entirely of bibrachial chromosomes constitutes a derived condition, apparently originated by pericentric inversions and heterochromatinization processes in some pairs. This chromosome morphology, considered structurally divergent from the basal pattern, in which acrocentric chromosomes prevail, appear to have contributed towards the reduction of the karyotypic diversification, frequently found in other species of Rivulidae.

Phylogenetic relationships derived from molecular data for Rivulidae suggest greater proximity of some species of the genus *Hypsolebias* (previously *Simpsonichthys*), with *Austrolebias* [45]. From the phylogenetic perspective, a more pronounced degree of karyotypic similarity would be expected, between *H. antenori* and *Austrolebias* species. In fact, the cytogenetic data of the species, as basal diploid value with 48 chromosomes, heterochromatin distribution largely restricted to centromeric regions, and the presence of a pair of chromosomes bearing ribosomal sites, shared with several species of *Austrolebias*, seem to support a close phylogenetic relationship with the genus [19, 38].

Despite the predictable shortcomings of cytogenetic information for some genera of the New World, the karyotypic survey for the species pertaining to 12 genera of South America reveals some common chromosomal patterns among them [14]. Thus, diploid values with 2n = 48 are present in most species of *Austrolebias* and *Kriptolebias*. Modal diploid numbers with >48 chromosomes appear to be a rare condition and are only found in one species of the genus *Aphyolebias* (2n = 54). Other genera reveal few species with 2n = 48 and most with 2n < 48, or just 2n < 48 fixed within their species.

The exclusive presence of bibrachial chromosomes in *H. antenori* should have possibly contributed to preventing numerical karyotypic changes and a low detectable change involving Ag-NORs sites. In fact, numeric polymorphisms or position of the inter or intraindividual Ag-NOR sites was not identified in this population. This contrasts with the high level of polymorphism of these chromosomal regions found in some species of Aplocheiloidei [39].

Cytogenetic analysis on eight putative species of *Austrolebias* identified a range between 3 and 6 active Ag-NORs sites. The heterochromatic regions in these species have been identified in telomeric, centromeric, and interstitial positions [19]. The absence of extensive translocation mechanisms as in some species of *Austrolebias* can contribute to the last position being absent in the chromosomes of *H. antenori* and also interstitial NORs. The combination of cytogenetic characteristics of *H. antenori* and species of *Austrolebias* suggest about ancestral states of some characters in chromosomes of the species/family. The extensive occurrence of multiple NORs present in two or three chromosome pairs seems to indicate that this is a symplesiomorphic condition for the family.

The data presented for *H. antenori* represent the first known information for this species in South America. Although the ribosomal sites are not polymorphic in *H. antenori*, the data available for several species of Rivulidae [14, 19, 38] indicate polymorphic condition and diversified sites, which are potentially useful as cytotaxonomic markers of populations.

Evolutionarily, karyotypic variations can represent efficient postzygotic barriers in killifish. Experiments of cross breeding between populations and species showed partial or complete reproductive isolation between cytogenetically different populations and species [41]. Chromosomal rearrangements, such as pericentric inversions, can cause cladogenesis events in populations of highly polymorphic killifish. Pattern recognition of cytogenetically divergent South American killifishes has been used to propose models of conservation in threatened species *Austrolebias* [38]. Analyses in new populations of *H. antenori*, as well as in other species of the genus, could establish the level of karyotypic divergence, as well as contributing information for biological conservation of this peculiar group of fish.

## 5. Conclusion

Killifishes are considered as intriguing biological models, due to their adaptive and ontogenetic peculiarities. Several groups within the suborder Aplocheiloidei have shown surprising karyotype diversification due to allopatric factors and possibly intrinsic characteristics of the karyotype. *Hypsolebias antenori*, an endemic fish from the semiarid region of Northeastern Brazil, presents a mixture of karyotypic patterns considered basal and derivative. Considering the extensive karyotype diversity in the family Rivulidae, the peculiar karyotypic data identified for the species, which constitute the first cytogenetic records for the genus, may provide a useful tool in the interpopulational analysis of variation and karyotype evolution of this group.
